# Alterations in Chromatin Structure and Function in the Microglia

**DOI:** 10.3389/fcell.2020.626541

**Published:** 2021-01-21

**Authors:** Yuki Fujita, Toshihide Yamashita

**Affiliations:** ^1^Department of Molecular Neuroscience, Graduate School of Medicine, Osaka University, Osaka, Japan; ^2^WPI Immunology Frontier Research Center, Osaka University, Osaka, Japan; ^3^Graduate School of Frontier Bioscience, Osaka University, Osaka, Japan; ^4^Department of Neuro-Medical Science, Graduate School of Medicine, Osaka University, Osaka, Japan

**Keywords:** brain, neuron, microglia, genome, development, chromatin 3D architecture

## Abstract

Microglia are resident immune cells in the central nervous system (CNS). Microglia exhibit diversity in their morphology, density, electrophysiological properties, and gene expression profiles, and play various roles in neural development and adulthood in both physiological and pathological conditions. Recent transcriptomic analysis using bulk and single-cell RNA-seq has revealed that microglia can shift their gene expression profiles in various contexts, such as developmental stages, aging, and disease progression in the CNS, suggesting that the heterogeneity of microglia may be associated with their distinct functions. Epigenetic changes, including histone modifications and DNA methylation, coordinate gene expression, thereby contributing to the regulation of cellular state. In this review, we summarize the current knowledge regarding the epigenetic mechanisms underlying spatiotemporal and functional diversity of microglia that are altered in response to developmental stages and disease conditions. We also discuss how this knowledge may lead to advances in therapeutic approaches for diseases.

## Introduction

Microglia are immune cells that have been studied extensively for their roles in pathological conditions. Microglia share many features with other substates of tissue-resident macrophages. Microglia respond rapidly to pathological stimuli via changes in morphology and function, such as releasing inflammatory cytokines, increased proliferation, and exhibiting active phagocytic properties (Ransohoff and Perry, 2009; Kettenmann et al., 2011; Shemer et al., 2015; Ransohoff, 2016). Advanced technologies, such as imaging and omics data analysis have identified roles for microglia that extend beyond their function as immune cells in physiological conditions. For instance, microglia communicate with neurons and survey the brain microenvironment, thus contributing to neuronal survival and maintenance of brain homeostasis (Tremblay et al., 2011; Wake et al., 2013). During brain development, microglia modulate diverse steps in the establishment of neural circuity, such as neuronal survival (Ueno et al., 2013; Fujita et al., 2020), axon outgrowth (Pont-Lezica et al., 2014; Squarzoni et al., 2014), and synaptic elimination (Hanisch and Kettenmann, 2007; Tremblay et al., 2011; Wake et al., 2013; Ueno and Yamashita, 2014; Mosser et al., 2017). These varied functions highlight the heterogeneity of microglia and their diverse responses and activities in both health and disease.

Histologically, microglia demonstrate regional heterogeneity (Tan et al., 2020). Microglia exhibit distinct morphologies and densities across different CNS regions in the healthy brain, which undergo alterations in disease or different stages of life. Microglia originate from yolk-sac macrophages (YSM) and enter the brain on embryonic day (E) 9.5 in mice (the timepoint at which neurons are first generated) (Casano and Peri, 2015; Ginhoux and Prinz, 2015; Prinz et al., 2017). Upon entering the brain, microglia expand and accumulate around white matter in the early postnatal brain, forming the “fountain of microglia” (Milligan et al., 1991; Monier et al., 2006; Hristova et al., 2010; Verney et al., 2010). These early microglia exhibit an amoeboid morphology, which differs from their ramified morphology in the adult brain (Milligan et al., 1991; Ling et al., 2001; Streit, 2001; Hristova et al., 2010; Ueno et al., 2013). In the adult brain, although most microglia possess ramified morphology with extended branches in physiological conditions, they exhibit unique morphology in certain brain regions. Compared to cortical microglia, microglia in the adult mouse cerebellum have less arbors and smaller somata (Verdonk et al., 2016; Stowell et al., 2018). With regards to density, the total number of microglia in the adult mouse brain is estimated to be ~3.5 × 10^6^, which is comparable to percentages of ~5% in the cortex and corpus callosum, and 12% in the substantia nigra of adult CNS cells (Lawson et al., 1990).

Microglia alter their gene expression profiles and characteristics in response to different conditions, highlighting their capacity for plasticity. Recent advances in transcriptomic analysis using bulk and single cell (sc)-RNA-seq have identified specialized substates of microglia across different CNS regions and contexts throughout developmental stages and various disease conditions (Hammond et al., 2019; Masuda et al., 2019; Sankowski et al., 2019). These coordinated gene expression profiles are underpinned by epigenetic modifications, including histone modifications such as acetylation, methylation, and phosphorylation; and DNA methylation. Epigenetic regulation is indispensable for normal brain development, and dysregulation of epigenetic states underscores disease pathology (Holtman et al., 2017; Cheray and Joseph, 2018). Alteration of epigenetic states often occurs in a context-dependent manner. For example, epigenetic mechanisms are involved in microglial function during development, disease, and reprogramming (Datta et al., 2018; Matsuda et al., 2019). In addition to epigenetic modifications in the linear genome, recent advanced technologies such as genome-wide chromatin analysis have revealed the importance of spatial chromatin architecture, such as chromatin loops that permit the association of gene promoters and other regulatory elements such as enhancers over short- and long-range linear genomic distances in transcriptional regulation (Dekker and Mirny, 2016; Szabo et al., 2019; Misteli, 2020).

This review focuses on the role of epigenetic mechanisms in the regulation of microglial heterogeneity and plasticity in physiological and pathological states. We discuss the potential involvement of microglial phenotypes and functions regulated by epigenetic modulators in neurodevelopmental pathologies and neurodegenerative diseases.

## Multilayered Organization of the Genome

**Figure 1:**
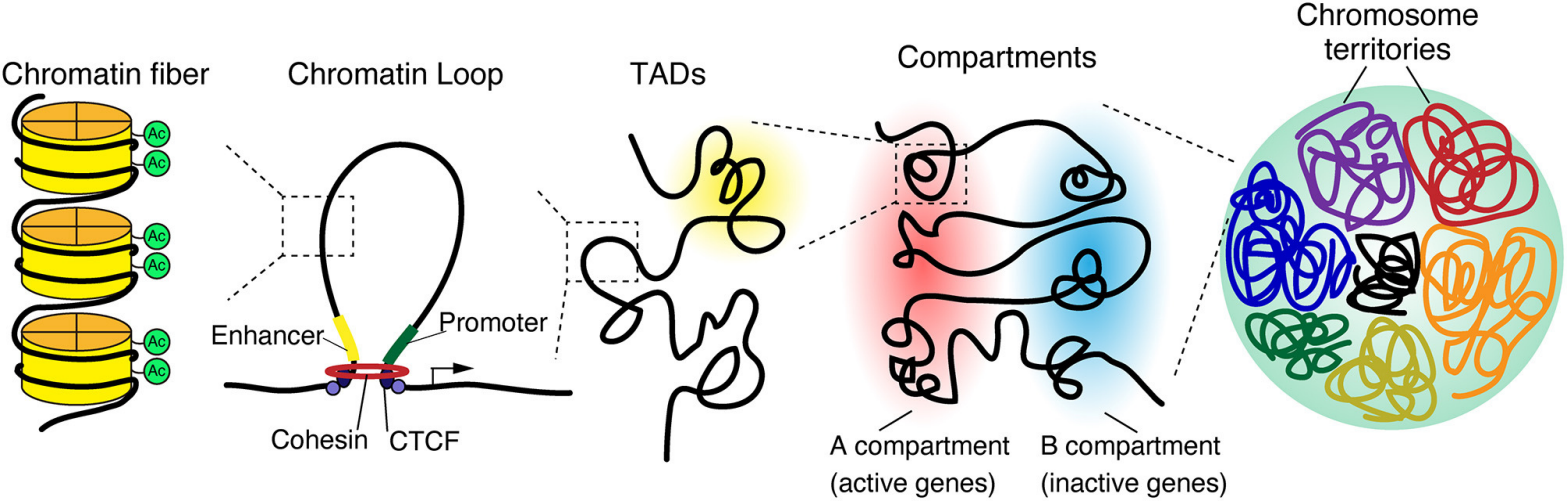
3D genome organization. The genome is organized in a hierarchical manner, starting at a nuclear level containing all chromosomes down to individual chromatin fibers. Chromosomes occupy distinct regions in the nucleus called chromosome territories and generally avoid overlap. Each chromosome is separated into A and B compartments that include the transcriptionally active or inactive genes, respectively. Both A and B compartments involve topologically associated domains (TADs), whereby the genomic associations strongly occur within the domain. TADs are generally bordered by CCCTC-binding factor (CTCF), which connects to linearly distant DNA sequences and brings them in close proximity, leading to the formation of a three-dimensional chromatin loop.

The epigenomic state of cells regulates gene expression, differentiation, and cellular identity (Crotti and Ransohoff, 2016; Holtman et al., 2017; Yeh and Ikezu, 2019). Recent technological advancements and genome wide analysis have identified the spatial structure of chromatin, including chromosome territories, A/B compartments, topologically associating domains, and chromatin loops, which are hierarchically organized in the three-dimensional nuclear space (Figure 1) (Phillips-Cremins, 2014; Bonev and Cavalli, 2016; Dekker and Mirny, 2016; Misteli, 2020).

**Figure 2:**
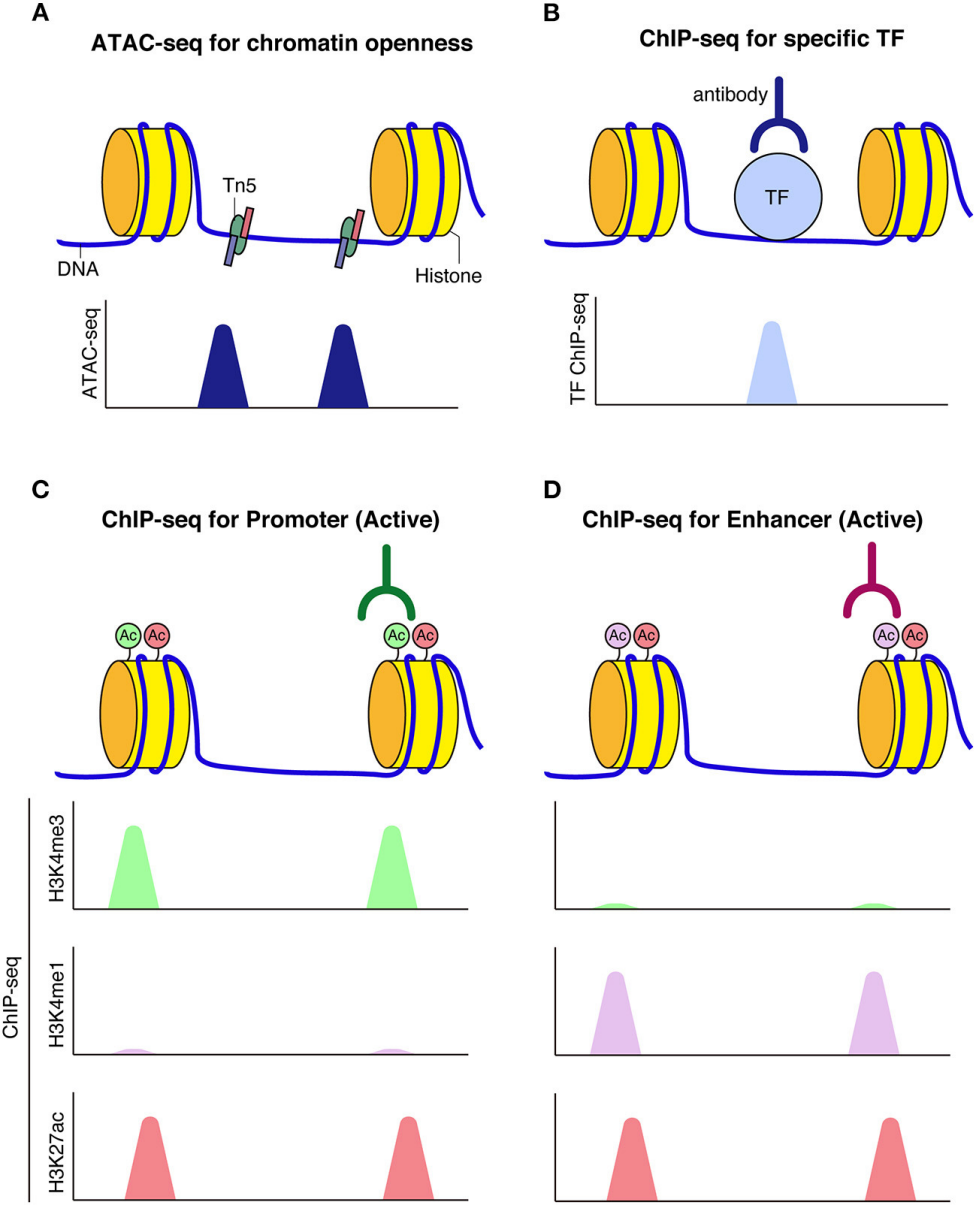
Schematic model of chromatin accessibility and histone marks at regulatory elements. **(A)** Assay for transposase-accessible chromatin using sequencing (ATAC-seq) can be used to capture chromatin openness, where Tn5 transposase simultaneously fragments and tags accessible DNA prior to sequencing. **(B)** When transcription factors bind to DNA, reads corresponding to TF bound fragments are obtained by sequencing. **(C,D)** Histone modifications mark functional genomic elements. Active enhancers are often marked by H3K27ac and H3K4me1 **(C)**. Active promoters are often marked by H3K27ac and H3K4me3 modifications **(D)**.

A series of genome-wide analyses have enabled the elucidation of higher-order chromatin architecture and histone modifications. The assay for transposase accessible chromatin (ATAC-seq) is a technique for identifying genome-wide accessible regions of chromatin based on transposase activity that inserts sequencing adapters into open regions of the chromatin (Buenrostro et al., 2013). Chromatin immunoprecipitation coupled with DNA sequencing (ChIP-seq) is a technique to identify protein-chromatin interactions by combining immunoprecipitation and high throughput DNA sequencing. ChIP-seq is widely used for the identification of cis-regulatory DNA elements, such as promoters, enhancers, and silencers, by targeting known histone modifications, transcription factors of interest, or proteins expected to be involved in enhancer activities, such as P300/CBP histone acetyltransferase (Simon and Kingston, 2009; Shlyueva et al., 2014; Andersson and Sandelin, 2020). Promoters and enhancers are typically marked by H3K4me3 and H3K4me1, respectively; both are additionally marked by H3K27ac upon activation. In contrast, silent or repressed promoters and enhancers are often marked by H3K27me3, which has been linked to Polycomb repression. Further, H3K9me3 typically labels transcriptionally silent heterochromatic regions (Figure 2).

A series of molecular techniques based on Chromosome-Conformation-Capture (3C), including 4C, 5C, and Hi-C, have been used to analyze the spatial organization of chromatin (Dekker et al., 2002; de Wit and de Laat, 2012). These methods enable the quantification of chromatin-chromatin interactions at different scales: 3C quantifies the interactions between two specific DNA fragments (one vs. one interactions) using PCR, whereas Hi-C quantifies the interactions between all possible pairs of fragments (all vs. all interactions) using paired end sequencing. These techniques have revealed that hierarchically organized spatial chromatin architecture is crucial for the regulation of gene transcription, which in turn is essential for the development and maintenance of various biological processes and epigenetic profiles of the linear genome such as histone modifications and DNA methylation (Phillips-Cremins, 2014). In the following sections, we summarize the current knowledge of the epigenetic profiles of microglia from these aspects and discuss their regulation and alterations in various contexts, including development, homeostasis, and disease.

## Genome Structure and Function of Microglia in Homeostasis

Even in normal conditions, microglia exhibit different transcriptional and epigenetic profiles. Macrophages populate all organs, and each population of tissue-specific macrophages, including microglia (brain-resident macrophages), is considered to adapt to its surrounding environment. Two groups have reported that cell type-specific enhancer and promoter landscapes regulate the identities of tissue-resident macrophages (Gosselin et al., 2014; Lavin et al., 2014). Enhancers are *cis* regulatory regions of DNA that allow the binding of multiple transcription factors to influence gene expression over variable distances, sometimes up to several hundred kilobases (kb) away. Chromatin loop formation enables the association with such distal enhancers to gene promoters (Sanyal et al., 2012; Shlyueva et al., 2014; Schoenfelder and Fraser, 2019). During development, the binding of lineage-specific transcription factors (TFs) to distinct enhancers is thought to be critical for the establishment of cell type-specific transcription by allowing local remodeling of chromatin and permanent accessibility to selective stretches of DNA (Heinz et al., 2015).

Tissue-resident macrophage populations have both common identities among general tissue macrophages and distinct enhancer profiles associated with the tissue specificity of macrophage subsets. Combination analysis of the chromatin landscape, including promoters (H3K4me3), poised enhancers (H3K4me1), and active enhancers (H3K27ac) of seven different tissue-resident macrophages, monocytes, and neutrophils with gene expression profiles and open chromatin regions revealed candidate tissue-specific transcriptional factors that contribute to shaping the chromatin specifications for tissue-resident macrophages (Lavin et al., 2014). In addition, transplant experiments revealed that the environment is partially capable of establishing the chromatin landscape of transplanted bone marrow precursors. Differentiated macrophages can be reprogrammed when transferred into a new microenvironment. Comparison of chromatin openness and transcriptomes between yolk sac-derived microglia and bone marrow graft-derived parenchymal brain macrophages revealed that graft-derived macrophages acquire microglial characteristics such as longevity, ramified morphology, and gene expression features but remain significantly distinct with respect to transcriptomes and chromatin accessibility landscapes (Shemer et al., 2018).

Furthermore, the brain environment also specifies gene expression in human microglia. Comparative studies of human and mouse microglial transcriptomes, including studies at the single cell level have revealed that human microglial gene expression correlates well with murine microglial gene expression, but numerous species-specific differences have been identified that include genes linked to neurological diseases in humans (Gosselin et al., 2017; Masuda et al., 2019). Transitions of human and mouse microglia from *ex vivo* brain tissue to an *in vitro* tissue culture environment resulted in remodeling of their respective enhancer landscapes alongside rapid and extensive down-regulation of genes that are induced in primitive mouse macrophages following migration into the fetal brain (Gosselin et al., 2017). Such environment-sensitive programs of gene expression and corresponding regulatory elements highlight the existence of dynamic transcription factor networks that underscore the identity and function of microglia. Collectively, these findings suggest that both specific factors associated with ontogeny and dynamic environmental factors cooperate to shape tissue-specific chromatin landscapes and gene expression profiles of macrophages.

Microglia also exhibit diversity in brain region-specific expression profiles and functions (Grabert et al., 2016; De Biase et al., 2017). Furthermore, environment-dependent epigenetic landscapes specify the gene expression profiles in both mouse and human microglia (Gosselin et al., 2017; Ayata et al., 2018). Cerebellar, but not striatal or cortical, microglia exhibit a high level of basal clearance activity associated with an elevated degree of cerebellar neuronal death in mice (Ayata et al., 2018). The microglia-specific translating ribosome affinity purification (TRAP) approach (Doyle et al., 2008; Heiman et al., 2008) permits region-specific analysis of microglia-enriched mRNA expression and precludes nonspecific microglia activation and concurrent upregulation of immediate early and inflammatory genes that occur during commonly used microglia isolation approaches. TRAP studies have revealed that cerebellar microglia exhibit cell-clearance phenotypes associated with exposure to dying cells. PRC2, which catalyzes the repressive chromatin modification histone H3 lysine 27 trimethylation (H3K27me3) (Margueron and Reinberg, 2011), epigenetically restricts the gene expression program that supports clearance activity in striatal and cortical microglia. H3K27me3 is absent from clearance-related gene loci in cerebellar microglia but not in striatal microglia. Loss of PRC2 leads to aberrant activation of clearance-specific genes in striatal microglia, which triggers changes in neuronal morphology and behavior, including decreased spine numbers in striatal medium spiny neurons (MSN) and MSN-mediated locomotor sensitization. These observations suggest that disturbances in epigenetic mechanisms are linked to aberrant activation of microglial clearance of neuronal damage and complex behavioral alterations associated with neurodegenerative and psychiatric diseases.

In normal conditions, microglia exhibit considerable heterogeneity across different CNS region (Tan et al., 2020). The epigenetic landscape of microglia varies among brain regions and may be associated with the maintenance of regional microglia specifications in the adult brain associated with their morphological and functional heterogeneity (Yeh and Ikezu, 2019). Microglia respond to environmental challenges, and their transcriptional epigenetic landscape can be dynamically altered in response to extrinsic stimulation. Therefore, the epigenetic landscape is specialized according to brain region, while retaining the capacity for plasticity and reprogramming (Holtman et al., 2017). A recent study demonstrated that the epigenetic regulation of microglia plays an important role in the reprogramming of microglia. Direct reprogramming of microglia into neurons has been achieved by expression of a single transcriptional factor, NeuroD1 (Matsuda et al., 2019), which has previously been used to convert astrocytes into neurons (Guo et al., 2014). The expression of NeuroD1 allows remodeling of the chromatin landscape from closed chromatin, associated with bivalent modifications (H3K4me3 and H3K27me3) to the monovalent mark (H3K4me3), associated with the establishment of neuronal identity at later stages of reprogramming. Concordant with pathological states, microglia accumulate and proliferate at injured sites and become the predominant cell type within the glial scar (Annunziato et al., 2013; Cregg et al., 2014). Therefore, modulating epigenetic and transcriptional profiles of existing microglia toward a neuronal phenotype may be a possible therapeutic approach to replenish lost neurons in CNS injury and disease.

## Genome Structure and Function of Microglia During Development and Cellular Differentiation

Microglia possess various epigenomic and associated transcriptomic signatures throughout life, including microglial development and aging. Studies with scRNA-seq have identified distinct spatiotemporal subpopulations of microglia with single cell resolution (Masuda et al., 2020b). Microglia demonstrate greater diversity during development, disease, and in the aging brain than in the normal, healthy adult brain (Hammond et al., 2019; Masuda et al., 2019; Sankowski et al., 2019). Genome-wide analysis of chromatin and expression profiles indicates that microglia undergo three distinct developmental stages, including early, pre-, and adult stages, with characteristic gene expression and functional states. Perturbations of this developmental process, such as knockout of the adult microglial transcription factor MafB, lead to disrupted brain homeostasis via the dysregulation of adult microglial genes and immune response pathways (Matcovitch-Natan et al., 2016).

A study of single-nucleus (sn) ATAC-seq in the mouse forebrain at seven developmental stages (E11.5, 12.5, 113.5, 14.5, 15.5, 16.5, P0) revealed chromatin accessibility profiles in microglia during development (Preissl et al., 2018). This study identified 12 distinct subpopulations of brain cells that exhibited abundant changes through development. Based on this classification, the chromatin accessibility profiles at gene loci of known marker genes have been addressed. The myeloid lineage cluster is restricted to E11.5 and disappears in later developmental stages. snATAC-seq data in the adult (P56) mouse forebrain identified one microglia cluster with accessibility at genes encoding complement factors, including the gene *C1qb*, leading to the inference that the adult forebrain comprises 6% microglia.

Dynamic developmental transitions of transcriptional and epigenetic profiles of human microglia throughout brain development have been analyzed (Schmunk et al., 2020). An integrative analysis of microglia, including transcriptomes, chromatin accessibility data generated using single cell ATAC-seq, and putative enhancer elements among open chromatin regions throughout human brain development has revealed the molecular signatures of stepwise maturation. Notably, human-specific cytokine-associated substates of microglia expressing increased levels of *C-C motif chemokine* (*CCL)2, CCL4*, and *interleukin* (*IL) 1B* are present in early brain development around the onset of neurogenesis. These findings demonstrate the dynamic transitions in transcriptional and epigenetic profiles in both mouse and human microglia. However, the molecular mechanisms underscoring the regulation of the epigenetic landscape are not fully understood.

### Sexual Dimorphism in Epigenetic Modulation of Microglia

Rodent microglia exhibit sexually dimorphic properties in pain perception, contribute to brain masculinization, and exhibit differences in brain colonization in males and females (Schwarz et al., 2012; Lenz and McCarthy, 2015; Mapplebeck et al., 2016). Furthermore, it was recently reported that microglia demonstrate transcriptomic differences in females and males throughout postnatal development (Hanamsagar et al., 2017). RNA-seq and ATAC-seq have revealed that microglia progressively gain sex-associated transcriptomic signatures and chromatin accessibility landscapes, which diverge in adult males and females (Thion et al., 2018). Microglia purified from female and male mouse brains at E18.5, shortly after the initiation of sex hormone production (Nelson and Lenz, 2017), exhibit low numbers of differentially expressed genes mostly present on the X and Y chromosomes, which may limit embryonic transcriptomic sexual dimorphism in adult females and males, consistent with other studies (Hanamsagar and Bilbo, 2017). In addition, female microglia display higher expression of genes associated with inflammatory responses, apoptotic processes, and responses to lipopolysaccharide (LPS). The absence of the microbiome in germ-free mice are more profoundly perturbed in the microglia of male embryos and female adults, highlighting the prenatal and postnatal impact of temporal and sexually dimorphic factors. ATAC-Seq has also revealed temporal changes in chromatin accessibility in the absence of the microbiome (Thion et al., 2018).

Several disorders exhibit sexual dimorphism. For instance, autism spectrum disorder (ASD) are more prevalent in males, whereas auto-immune diseases are more prevalent in females (McCarthy and Wright, 2017; Nelson and Lenz, 2017). These findings underscore the need to identify how transcriptomic and epigenetic sexual dimorphism in microglia is associated with their differentiation or functional differences linked to CNS diseases. Further work using animal models of these diseases should address the effects of temporal and sexually dimorphic factors in modulating the epigenetic and transcriptomic landscape of microglia in disease onset and progression.

### Genome Dynamics During Cellular Differentiation

Cell fates are specified by lineage-determining transcription factors. Epigenetic mechanisms regulate lineage-determining transcription factors which bind to genomic regions in a cell-specific manner. Macrophages and B cells play essential and complementary roles in the innate and adaptive arms of the immune system. Within the mammalian hematopoietic system, these cell types are derived from a lymphoid-primed multipotential progenitor (LMPP) that subsequently gives rise to common lymphoid progenitors (CLPs). CLPs differentiate into B cells or granulocyte-macrophage progenitors (GMPs) cells that can differentiate into macrophages (Adolfsson et al., 2005). ChIP-seq revealed distinct PU.1 binding patterns within the vicinity of motifs bound by lineage-restricted transcription factors in macrophages and B cells, respectively (Heinz et al., 2010). PU.1 binding induces nucleosome remodeling followed by H3K4 monomethylation (H3K4me1) that may signify accessible chromatin and/or enhancer-like elements (Heintzman and Ren, 2009) at large numbers of genomic regions associated with both broadly and specifically expressed genes. PU.1-bound sites in macrophages also play a role in shaping the transcriptional response to inflammatory stimuli such as LPS, likely by generating cell type-specific regions of open chromatin that allows the recruitment of transcriptional coactivators (Ghisletti et al., 2010). These findings provide insight into the extensive genome-wide and cell type-specific colocalization of transcriptional factors.

A study using the Hi-C approach, which is a genome-wide approach for the detection of interactions between all mappable regions of entire chromatin, has revealed the three-dimensional chromatin arrangement and transcription during cellular differentiation from human monocytes and differentiated macrophages (Phanstiel et al., 2017). This study employed a model using the monocytic leukemia cell line THP-1 treated with phorbol myristate acetate (PMA), which is widely used for studying the differentiation from monocytes to macrophages and provides an ideal system for studying the regulatory dynamics of long-range interactions (Daigneault et al., 2010). A modified Hi-C method, *in situ* Hi-C, permits higher resolution and unbiased genome-wide detection of DNA loops. Compared to static (pre-formed) loops, acquired loops during macrophage differentiation are enriched for H3K27ac, consistent with enhancer activity and gene promoters. Enhancer-bound loop formation and enhancer activation of preformed loops form multiloop activation hubs at key macrophage genes during macrophage development. Each multiloop activation hub in differentiated macrophages involves the interaction of on average 3.4 enhancers to a promoter and exhibits a strong enrichment of the binding sites for activator protein 1 (AP-1), a key transcriptional regulator for the differentiation of monocytic precursors into mature macrophages. These findings suggest that the distal regulation of gene transcription mediated by DNA loops, which bring enhancers in close proximity to their target genes, represents a major mechanism for controlling the developmentally regulated expression of distinct genes.

**Figure 3:**
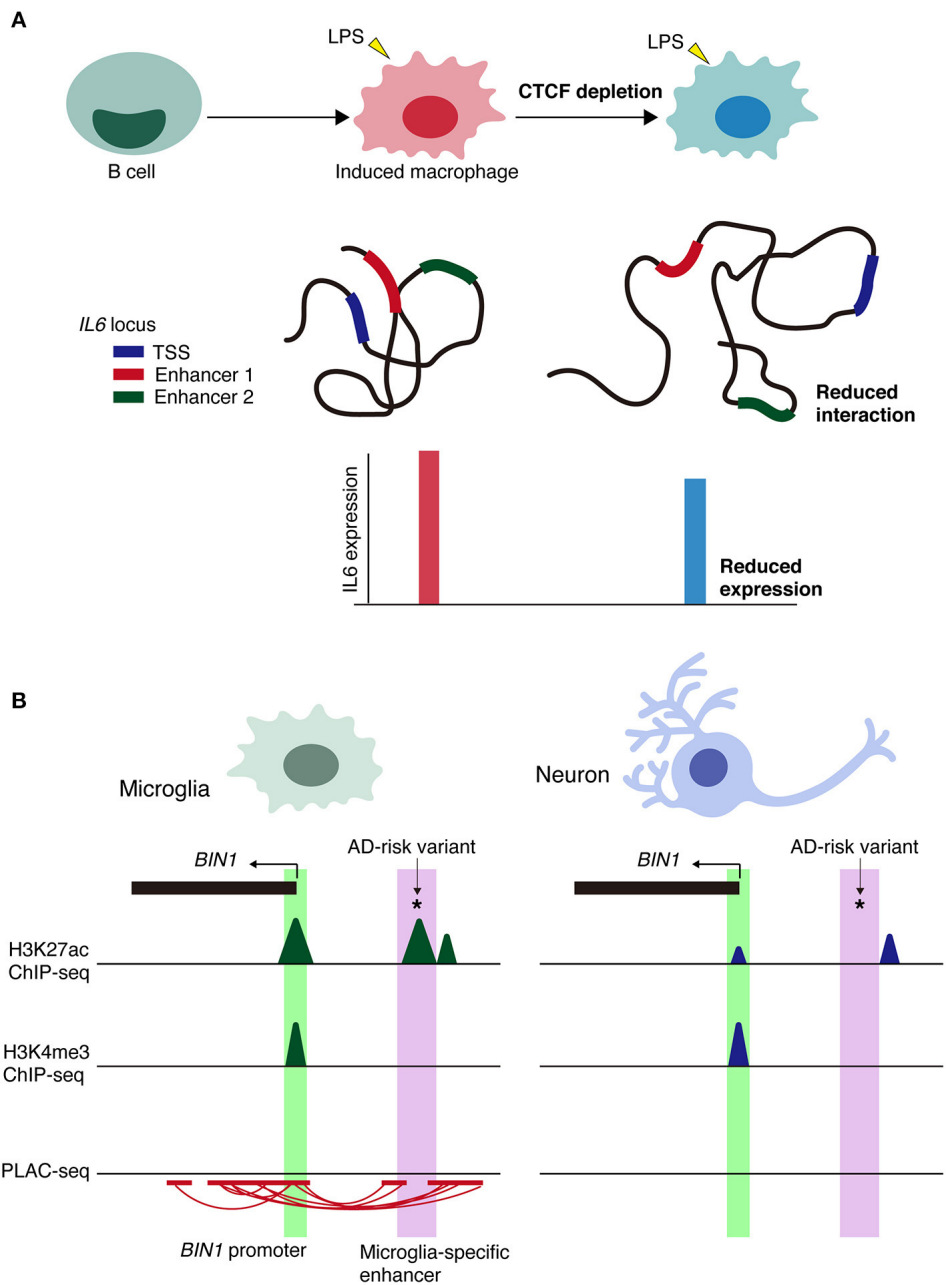
Context-dependent alterations of chromatin interactions. **(A)** Schematic model of the alterations in chromatin architecture and the impairment of inflammatory responses of CTCF depletion in immune cells via lipopolysaccharide (LPS) stimulation. Loss of CTCF reduces the frequency of chromatin interactions, such as enhancer-promoter interactions at *IL6* locus. TSS, transcription start site. **(B)** Schematic representation of microglia-specific enhancer region (highlighted in purple) harboring AD-risk variants at *BIN1* loci. Active promoter regions (highlighted in green) are shared between cell types. PLAC-seq demonstrates that microglia-specific enhancers are linked to the *BIN1* promoter. *Indicates AD-risk variant.

A recent study showed that the spatial architecture of chromatin is important for inflammatory response rather than the differentiation of immune cells (Stik et al., 2020). CCCTC-binding factor, CTCF binds to DNA and are involved in the formation of TADs and long-range chromatin loop (Phillips and Corces, 2009). CTCF depletion disrupts TAD organization but did not affect the differentiation of human leukemic B cells into macrophages (Stik et al., 2020). In contrast, CTCF depletion in induced macrophages impairs the upregulation of inflammatory genes upon lipopolysaccharide (LPS) stimulation and decreased the frequency of the enhance-promoter interaction at the *IL6* locus (Figure 3A).

Although the dynamic alterations of spatial chromatin organization have been addressed, the causes or consequences of 3D genome dynamics still remain unclear. In addition, analyses of microglia from this aspect are lacking, which should be addressed by future studies.

## Genome Structure and Function of Microglia in Pathological Conditions

Microglia contribute to various processes including brain development and homeostasis throughout the lifespan. Microglia regulate early wiring, synaptic pruning and formation, and cell death and survival, which are indispensable for establishing and maintaining neural circuits (Ransohoff and El Khoury, 2015; Schafer and Stevens, 2015; Hong et al., 2016; Tay et al., 2017; Thion and Garel, 2017; Wolf et al., 2017). Consistent with their diverse roles, microglia have been linked to the initiation or progression of several developmental and neurodegenerative diseases, including ASD, schizophrenia, Alzheimer's disease (AD), Parkinson's disease (PD), and multiple sclerosis (MS) (Shemer and Jung, 2015; Colonna and Butovsky, 2017). As microglia possess different epigenomes and associated transcriptomes throughout the life course, perturbations of epigenetic regulation may result in diverse effects that may underscore disease onset and progress.

### Alzheimer's Disease

Reactive microglia are associated with almost all neurodegenerative diseases (Streit et al., 1999). Reactivity of microglia and elevated cytokine levels are observed in the brains of AD patients. Nevertheless, the mechanisms underlying microglial activation and their contribution to disease progression remain poorly understood. Both transcriptomic and proteomic analysis at the single cell level have revealed the entire immune landscape and different expression profiles in CNS pathology (Keren-Shaul et al., 2017; Mathys et al., 2018; Mrdjen et al., 2018). Depletion of both histone deacetylases Hdac1 and Hdac2 in microglia result in different effects in the developing, homeostatic, and diseased brain (Datta et al., 2018). Hdac1 and Hdac2 are essential for microglial survival during brain development in mice but not during homeostasis in adulthood. In 5xfamilial AD (5xFAD) transgenic mice (an AD mouse model), deletion of microglial Hdac1 and Hdac2 enhances microglial phagocytosis of amyloid plaques and improves cognitive function.

In addition, ten-eleven translocation 2 (TET2) methylcytosine dioxygenase is expressed by amyloid beta (Aβ) plaque-associated microglia in brain tissue in both 5xFAD mice and individuals with AD (Carrillo-Jimenez et al., 2019). TET2 is involved in early gene transcriptional changes, leading to early metabolic alterations, and later inflammatory responses independently of its enzymatic activity. TET2 is upregulated in microglia upon exposure to inflammatory stimuli via an NF-kB-dependent signaling pathway, which involves epigenetic mechanisms. Following inflammatory stimulation, the level of H3K27ac marking increases at the Tet2 promoter and upstream regions concomitant with the recruitment of p65 to both the promoter and upstream regions.

Injection of interleukin (IL)-33 in APP/PS1 mice (an amyloid-deposition mouse model) ameliorates Aβ pathology by reprogramming microglial epigenetic and transcriptomic profiles to induce a microglial subpopulation with enhanced phagocytic activity (Lau et al., 2020). IL-33 enhances microglial Aβ clearance by inducing a subpopulation of major histocompatibility complex class II (MHC-II)-positive phagocytic microglia, which in turn are regulated by PU.1-dependent transcriptome reprogramming. ATAC-seq and ChIP-seq analysis revealed that IL-33-induced remodeling of chromatin accessibility and transcription factor PU.1 binding at the signature genes of IL-33-responsive microglia regulate their transcriptome reprogramming. Thus, IL-33-induced epigenetic and transcriptional regulation of microglial state transitions contributes to the alleviation of AD pathology. Although effective therapies for AD are currently lacking, these findings provide novel insight into the therapeutic potential of reprogramming the epigenetic and transcriptome profiles of microglia to treat AD.

Context-specific microglial phenotypes have been reported, including disease-associated microglia (DAM) (Keren-Shaul et al., 2017) and the microglial neurodegenerative phenotype (MGnD) (Krasemann et al., 2017). Comparison of DAM enhancers in wildtype (WT) and 5xFAD mice using a high sensitivity method for ChIP-seq analysis (iChIP) (Lara-Astiaso et al., 2014) revealed a similar level of H3K4me2, which marks promoter and enhancer regions (Keren-Shaul et al., 2017). These findings suggest that the disease-associated regions primed in DAM are already primed in homeostatic microglia.

Innate immune memory is a key mechanism underlying myeloid cell plasticity that occurs in response to environmental stimuli (Netea et al., 2015, 2016). This mechanism can be classified into immune training, which enhances immune responses to subsequent immune insults, and immune tolerance, which suppresses inflammatory responses to subsequent stimuli (Biswas and Lopez-Collazo, 2009; Cheng et al., 2014; Saeed et al., 2014). In a mouse model of AD, cerebral β-amyloidosis is exacerbated by immune training and alleviated by immune tolerance via epigenetic modifications (Wendeln et al., 2018). ChIP-seq analysis revealed that increased H3K4me1 levels in microglia from 1xLPS (immune training) vs. 4xLPS (immune tolerance) WT animals exhibited enrichment for the thyroid hormone signaling pathway, including a putative enhancer for hypoxia inducible factor-1a (HIF-1a). Similar results were observed in AD model mice (APP23) injected with 1xLPS vs. 4xLPS (Wendeln et al., 2018). In addition, microglia from 4xLPS-treated AD model mice demonstrated increased H3K4me1 levels in putative enhancers related to phagocytic function. These observations highlight the differential effects of immune training vs. tolerance due to multiple environmental stimuli, which is reflected in the epigenetic landscape of DAM and/or MGnD in AD mouse models. Further studies should elucidate how environmental stimulation modulates the epigenetic landscape for context-specific microglial functions and their contribution to the progression of neurodegenerative disorders.

Comprehensive studies of the transcriptional and epigenetic landscapes of isolated microglia from human and mouse brain tissue samples using RNA-seq, ChIP-seq, and ATAC-seq have revealed the involvement of microglia in disease mechanisms (Gosselin et al., 2017; Tansey et al., 2018; Nott et al., 2019). The transcriptional profiles of cortical microglia defined 881 transcripts as the unique microglial gene signature (Gosselin et al., 2017). This core transcript set was compared with 46 publicly available microarray or RNA-seq datasets of genes that are differentially regulated in neurodegenerative and behavioral disorders. Of these, 28 exhibited enrichment or depletion of the microglial signature. More than half of the genes associated with noncoding genome-wide association study (GWAS) risk alleles for AD and MS are preferentially expressed in microglia. In contrast, fewer genes associated with PD and schizophrenia risk alleles exhibited preferential expression in microglia. These findings underscore the diverse roles of microglia in the context of different diseases.

Alongside transcriptional alterations, perturbations in gene expression regulation are inferred to be key mechanisms since the majority of disease-associated genetic variation resides in non-coding regions of the genome (Maurano et al., 2012; Khurana et al., 2016). To better understand genetic variation associated with brain diseases, isolated nuclei from different brain cell types, including neurons, astrocytes, microglia, and oligodendrocytes from cortical brain tissue of human individuals were subjected to ATAC-seq to assess open chromatin regions and ChIP-seq for H3K27ac and H3K4me3 to address active enhancers and promoters, respectively, in each brain cell type (Nott et al., 2019). Whereas, active promoters were mostly common across cell types, the fraction of active enhancers that overlapped between different cell types was small, suggesting that cell type specificity is modulated predominantly by the enhancer repertoire.

Linkage disequilibrium score (LDSC) regression analysis can be utilized on GWAS summary statistics to determine SNP-based genetic heritability for a trait or disease. Psychiatric disorders or behavioral traits are primarily associated with variants in transcriptional enhancers and promoters in neurons. In contrast, sporadic AD risk variants are largely confined to microglial enhancers. To detect long-range chromatin interactions at the promoter region, H3K4me3 proximity ligation-assisted ChIP-seq (PLAC-seq), in which proximity ligation is conducted in nuclei prior to chromatin shearing and immunoprecipitation (Fang et al., 2016), has been performed. This method revealed 219,509 significant interactions across cell types (Nott et al., 2019). Interactome maps from PLAC-seq identified several parameters, including: (1) AD-risk variants that were linked to more distal active promoters and not the closest promoter; (2) enhancers harboring AD-risk variants that were PLAC-linked to active promoters of both GWAS-assigned genes and an extended subset of genes not assigned to GWAS loci; and (3) cell type-specific enhancers harboring AD risk variants linked to genes expressed in multiple cell types, suggesting cell type-specific disease susceptibility.

Regarding cell type-specific enhancers, the *BIN1* microglia-specific enhancer is PLAC-linked to the *BIN1* promoter and harbors the AD-risk variant rs6733839, which has the second highest AD-risk score (Figure 3B). Deletion of a microglia-specific enhancer harboring AD-risk variants ablated *BIN1* expression in microglia but not in neurons or astrocytes. Collectively, these findings demonstrate the value of chromatin interactome maps to the functional interpretation of GWAS risk alleles associated with neurological and psychiatric diseases. Alterations in cell type-specific enhancer-promoter interactions may be a prominent mechanism underlying genetic variants in non-coding regions associated with disease onset and/or progression.

### Huntington's Disease (HD)

HD is a neurodegenerative disorder caused by specific expansion of a CAG repeat in the coding region of the *HTT* gene (The Huntington's Disease Collaborative Research Group, 1993). Similar to neurodegenerative diseases such as AD and PD, reactive microglia and elevated cytokine levels are observed in the brains of both mice and humans with HD (Sapp et al., 2001; Tai et al., 2007). Genome-wide approaches including RNA-seq and ChIP-seq have revealed that the expression of mutant Huntingtin (mHTT) in microglia but not in bone marrow-derived macrophages causes cell autonomous pro-inflammatory transcriptional activation through increased expression and transcriptional activity of myeloid lineage-determining factors PU.1 and C/EBPs (Crotti et al., 2014). ChIP-seq analysis for PU.1 and H3K4me2, a histone modification associated with enhancers and promoters (Regha et al., 2007; Brykczynska et al., 2010; Chepelev et al., 2012), demonstrated that genomic loci encoding mRNAs that are upregulated in BV2 microglia expressing mHTT generally exhibit higher enrichment of PU.1 binding to promoters/enhancers, exemplified by the Tnf locus. Similar results were observed in ChIP-seq analysis for C/EBPα and C/EBPβ. The binding sites for PU.1 and C/EBPs are highly enriched in enhancers and promoters associated with genes exhibiting constitutive upregulation in mHTT-expressing microglia. Collectively, these observations indicate that disruption of epigenetic and transcriptomic regulation in microglia affects neuronal function. Deeper understanding of the effects of microglial identity on interactions with neurons will provide further insight into the contribution of microglial activation to the pathophysiology of neurodegenerative diseases such as AD and HD.

### Rett Syndrome

Aberrant epigenetic regulation in microglia is also implicated in neurodevelopmental and psychiatric disorders. Growing evidence suggests that alterations in spatial chromatin structure is associated with neurodevelopmental and neuropsychiatric disorders. Rett syndrome is an ASD caused primarily by mutations in methyl-CpG binding protein 2 (MeCP2) (Amir et al., 1999) and is characterized by prominent neurologic dysfunction. Accordingly, efforts to understand the function of MeCP2 have largely focused on its role in neurons (Chahrour and Zoghbi, 2007). More recently, the expression and roles of MeCP2 in astrocytes (Ballas et al., 2009; Lioy et al., 2011; Yasui et al., 2013), oligodendrocytes (Nguyen et al., 2013), and microglia (Maezawa and Jin, 2010; Derecki et al., 2012) have been reported. Microglia of MeCP2-null mice, a mouse model of Rett syndrome, exhibit reduced phagocytic activity. Transplantation of MeCP2-null mice with WT microglia ameliorates disease progression, suggesting that the phagocytic properties of microglia are indispensable for normal brain development and function, and deficits in microglial phagocytosis may be associated with disease onset and/or progression. In addition, ChIP-seq analysis revealed that MeCP2 deletion increased histone H4 acetylation at enhancer regions of *Fkbp5* (a canonical glucocorticoid target gene) and recruitment of nuclear receptor corepressor 2 and HDAC3 complex (Cronk et al., 2015). Thus, MeCP2 deletion resulted in the upregulation of *Fkbp5* gene expression thorough epigenetic mechanisms, suggesting that MeCP2 deletion underpins microglial dysfunction in Rett syndrome. In contrast, another study reported that wild-type microglia or specific Mecp2 expression in microglia did not rescue the pathology in Mecp2 null mice (Wang et al., 2015). The contribution of the microglia in Rett syndrome and the therapeutic potential of targeting the microglia in this disease are still being debated.

### Pain

Neuropathic pain is a chronic and devastating condition that occurs following nerve damage or in various diseases (Basbaum et al., 2009). Animal studies have demonstrated that characteristic changes in both neurons, glial cells, and neuro-glial interactions, play a key role in the establishment and maintenance of persistent pain (Tsuda et al., 2003; Calvo and Bennett, 2012; Denk et al., 2016). Genome-wide transcriptional profiles of isolated spinal cord microglia following partial sciatic nerve ligation, which is a widely used model of neuropathic pain, have been identified by RNA-seq. H3K4me1 ChIP-seq analysis revealed injury-induced alterations in microglial enhancer profiles, possibly associated with transient transcriptional upregulation (Denk et al., 2016). Although the time-course analysis for expression changes revealed that transcriptional upregulation reverts to baseline by 28 days following pain induction, ChIP-qPCR identified several putative latent enhancer regions with increased H3K4me1 binding levels up to a month following pain induction. These findings reveal persistent injury-specific alterations of the microglial enhancer landscape.

## Discussion

Based on cell type-specific isolation of microglia and/or techniques using deep sequencing, epigenetic and transcriptomic profiles of microglia have been identified. However, much remains unknown of their causal or consequential effects, such as the regulation of functional, morphological, and regional heterogeneity of microglia in a context-dependent manner. Animal models with inducible drivers of Cre-recombinase such as Cx3cr1-CreER (Yona et al., 2013) allow the use of microglia-targeted tracing and microglia-specific knockout or overexpression of genes of interest. One study reported that several substates of microglia downregulate CX3CR1, limiting this model's utility in the study of microglia (Stratoulias et al., 2019). Recent studies have highlighted that the CX3CR1 line targets the microglia and CNS border-associated macrophages (Goldmann et al., 2016; Chappell-Maor et al., 2020). Newly developed mouse lines that express inducible Cre or fluorescence reporter genes specifically in the microglia, such as the Trem119-CreERT2 (Kaiser and Feng, 2019), Hexb-CreERT2 (Masuda et al., 2020a) and P2ry12-CreER (McKinsey et al., 2020) transgenic mice would be powerful tool in further studies. These approaches will help address the functional roles of epigenetic changes in microglial regulation and the manner in which they affect the nervous system *in vivo*.

A further step forward is to decipher how epigenetic profiles, especially spatial structures of chromatin, respond to various environmental cues. Microglia are exposed to diverse cues depending on developmental and pathological context. Even under normal conditions, microglia exhibit altered epigenetic marks. However, the influence of environmental cues on microglial physiological identity and disease-specific responses remains elusive. In addition, not all microglia respond to certain cues, which implies different substate-dependent susceptibility. There exists a unique microglial phenotype, known as the dark microglia, which is identified by the alteration in nuclear chromatin at the ultrastructural level (Bisht et al., 2016). This substate is rarely found in the physiological state in some areas of the brain, including the hippocampus, cerebral cortex, amygdala, and hypothalamus. However, they proliferate in conditions such as chronic stress, aging, fractalkine signaling deficiency, and Alzheimer's disease pathology. These observations suggest the association between the chromatin structure of microglia and their roles in the pathological remodeling of neuronal circuits. Advancements in technologies to elucidate transcriptomes, chromatin accessibility, and the interactions between DNA at a single cell level, including single cell RNA-seq, single cell ATAC-seq, and single cell Hi-C, will help to overcome the aforementioned technical limitations.

Recent studies have identified disturbances in enhancer-promoter interactions in the diseased brain (Rajarajan et al., 2018; Nott et al., 2019). In addition, deletion of regulatory proteins for chromatin loop formation is associated with deficits in synapse formation during brain development and behavioral deficits (Hirayama et al., 2012; Fujita et al., 2017). In this regard, it is plausible that aberrant changes in 3D chromatin structure contribute to neurodevelopmental diseases. Although the mechanisms by which epigenetic marks and changes in spatial chromatin structure regulate microglial function are largely unknown, elucidating these mechanisms will provide a step forward in understanding the role of microglia in neurodevelopmental and neuropsychiatric disorders. Studies in animal models and humans, including tissues and cells derived from induced pluripotent stem cells of patients with relevant diseases will provide novel insight into the role of microglia in disease pathogenesis.

## Author Contributions

YF wrote the manuscript, and TY revised it. All authors contributed to the article and approved the submitted version.

## Conflict of Interest

The authors declare that the research was conducted in the absence of any commercial or financial relationships that could be construed as a potential conflict of interest.
